# Paging Dr. Google: Characterizing Direct-to-Consumer Digital Advertisements From Oncology Treatment Centers

**DOI:** 10.7759/cureus.80994

**Published:** 2025-03-22

**Authors:** Katie M Huisman, Mason K Whitaker, Skyler B Johnson, Gary V Walker

**Affiliations:** 1 College of Medicine, University of Arizona College of Medicine - Phoenix, Phoenix, USA; 2 School of Life Sciences, Arizona State University, Tempe, USA; 3 Department of Radiation Oncology, University of Utah Huntsman Cancer Institute, Salt Lake City, USA; 4 Department of Radiation Oncology, Banner MD Anderson Cancer Center, Gilbert, USA

**Keywords:** accreditation, advertisements, cancer treatment center, commission on cancer, direct-to-consumer marketing, national cancer institute, oncology

## Abstract

Purpose: This study aims to examine the characteristics and accreditations of medical centers with the highest utilization of direct-to-consumer (DTC) marketing based on Google search advertisements.

Methods: A total of 817 paid advertisements from 360 simulated Google searches from the 30 most populous U.S. cities were analyzed. They were categorized into National Cancer Institute (NCI)-designated, Commission on Cancer (CoC) accredited, non-CoC accredited, and non-traditional treatment centers and analyzed based on city, region, and search term.

Results: Of the cancer treatment center advertisements analyzed, 51.2% were for NCI centers, 12.2% for CoC accredited centers, 26.9% for non-CoC accredited centers, and 9.7% for non-traditional centers. There was significant geographic variation, with the highest percentage of NCI advertisements appearing for the search phrase “best cancer doctor” (74.4%). The majority of NCI centers had no advertisements (59.2%), while two of the centers monopolized the NCI advertisements (80.2%). Non-traditional treatment centers showed significant geographic variation, representing 0-38% of search results.

Conclusion: The study reveals a significant presence of NCI-designated cancer centers in the digital DTC marketing space, with substantial regional variations and a disproportionate number of advertisements from a minority of cancer centers.

## Introduction

Direct-to-consumer (DTC) marketing in healthcare has been increasing since the early 2000s [1]. Cancer remains the second-leading cause of death, with approximately two million new cancer diagnoses annually in the United States [2].

There is evidence exploring aspects of DTC marketing by cancer centers, including expenditures and the most frequently used platforms [1,3,4]. These studies highlight digital marketing as the fastest-growing DTC platform in terms of advertising expenditures [3]. Digital marketing often gives users a sense of control over their online searches, but these searches are not unbiased [5]. Cancer patients researching treatment options are likely to encounter and be influenced by those who have paid for prominent ad placements. Studies have examined tactics and ethical implications of cancer treatment marketing [4,6,7], yet a noticeable literature gap remains regarding the characterization of treatment centers that contribute to online DTC marketing. 

The purpose of this study is to investigate the characteristics and accreditations of the medical centers with the highest utilization of DTC marketing based on Google searches. This project was previously presented as a poster at the ASTRO Annual Meeting in October of 2023.

## Materials and methods

A series of keyword searches were conducted using the Google search engine on the Google Chrome browser. Search trends were investigated by using the Google Trends^TM^ tool, which provides search volumes based on popular search queries. Three of the top relevant search phrases were identified (“best cancer doctor”, “best cancer treatment”, and “cancer treatment near me”). Modifiers were added to each search term to reflect cancer diagnoses with the highest prevalence: “breast”, “prostate”, and “lung”. This yielded 12 distinct search phrases that were used on a search hub (I Search From) that simulated Google searches from different geographic locations [8]. 

**Figure 1:**
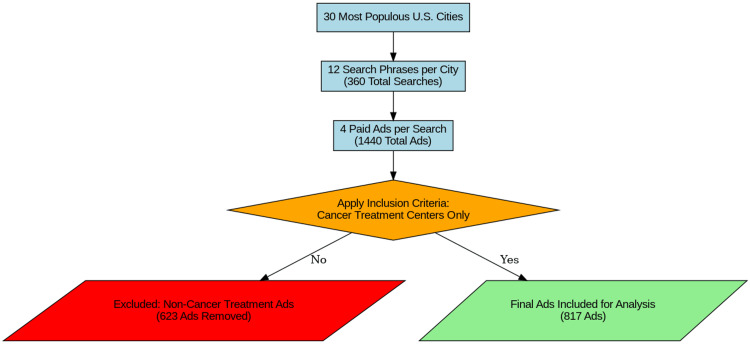
Flowchart depicting the study’s methodology

The 30 most populous U.S. city locations were used for each of the 12 search phrases to originate the searches, resulting in a total of 360 searches conducted. Each search yielded four paid advertisements which appear at the top of the results list, totaling 1,440 total advertisements. The inclusion criteria for analysis were advertisements related to cancer treatment centers. Advertisements for non-cancer center treatments (i.e., pharmacologic or other therapies) were excluded, with 623 advertisements falling under the exclusion criteria. After applying these criteria, 817 advertisements were included in the study for analysis. A flowchart of the above methodology is depicted in Figure 1.

**Figure 2:**
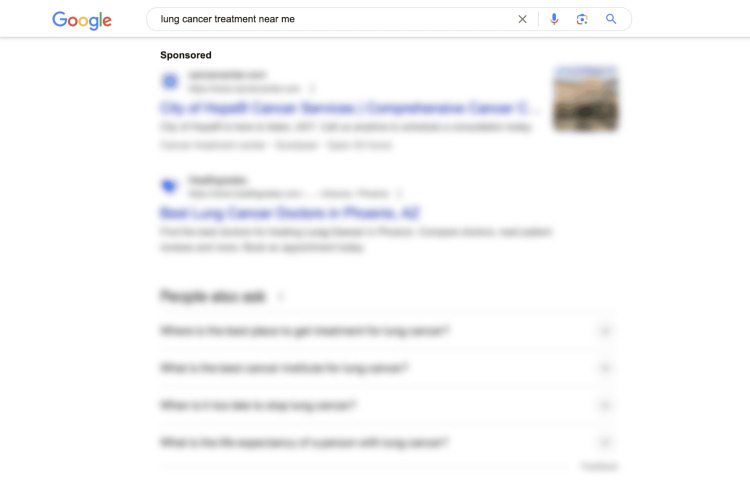
“Sponsored” label used to indicate results that represent paid advertisements

An example of the paid advertisements labeled as “sponsored” is shown in Figure 2.

Of the advertisements, the cancer treatment centers were categorized into centers that are National Cancer Institute-Designated (NCIs) [9], Commission on Cancer (CoC) accredited [10], non-CoC accredited, and non-traditional treatment centers based on their category at the time of the search. Note that NCI and CoC are the major accreditation agencies in regard to cancer care in the United States. In addition, non-traditional treatment centers were defined as facilities that incorporate unconventional approaches to cancer care, often referred to as integrative, complementary, or alternative therapies [11]. The searches were conducted in December of 2022. 

The statistical analysis and data aggregation were conducted using Microsoft Excel (version 2208, Microsoft Corp., USA). This software was utilized for all data analysis tasks, including organizing and analyzing the collected data. No additional statistical software was employed.

## Results

**Figure 3:**
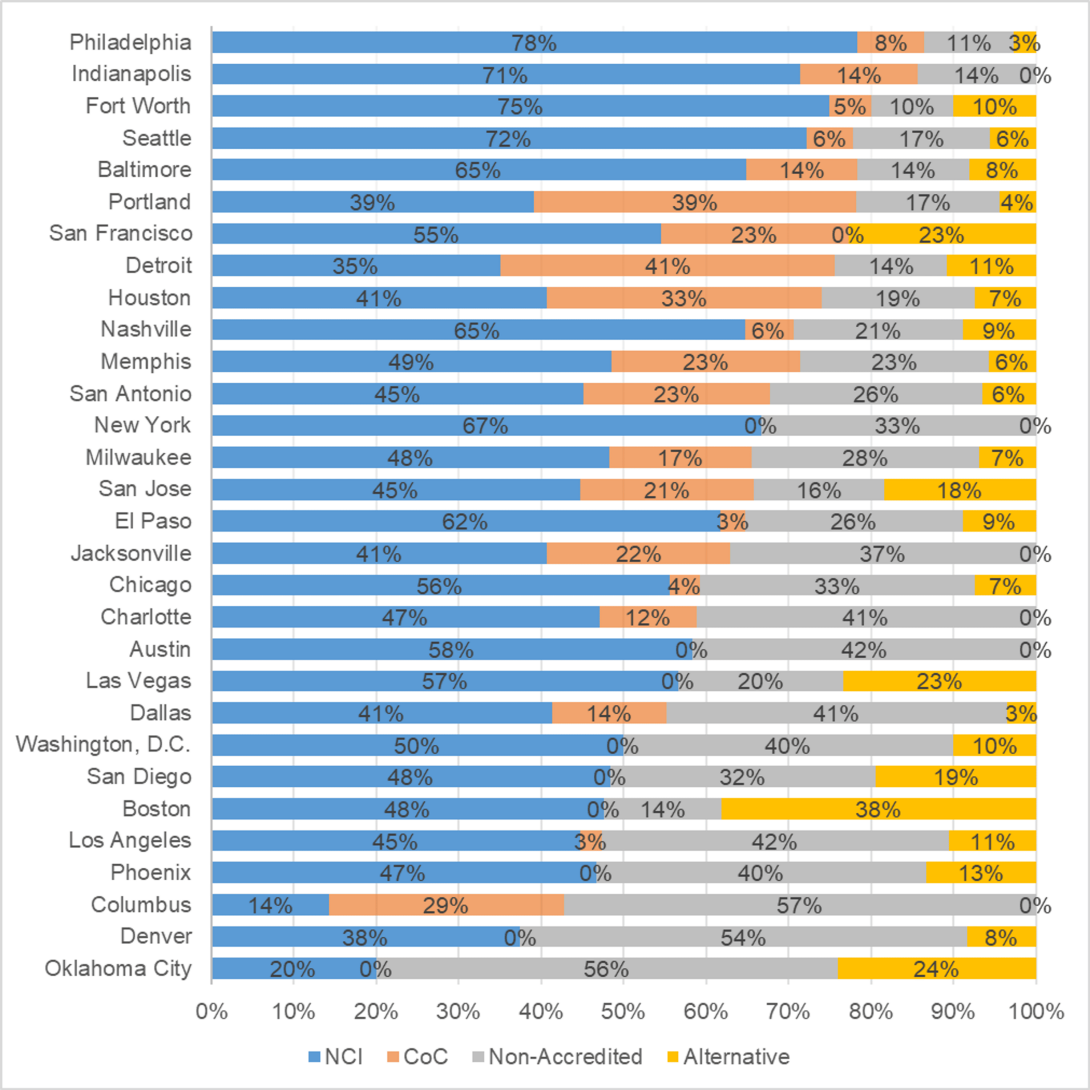
Percentage of search results that were National Cancer Institute (NCI)-accredited centers, Commission on Cancer (CoC)-accredited centers, non-accredited centers, and non-traditional centers based on city and sorted by the highest percentage of accredited centers

Of the 360 searches and 817 subsequent cancer treatment center advertisements, 51.2% were for NCI centers, 12.2% were CoC accredited centers, 26.9% were non-CoC accredited centers, and 9.7% were non-traditional treatment centers. The proportions of treatment center advertisements by city that were for NCI-accredited centers ranged from 14.3% to 78.4%, CoC-accredited centers ranged from 0.0% to 40.5%, non-accredited centers ranged from 0% to 57.1%, and non-traditional centers ranged from 0% to 38.1%. (Figure 3).

**Figure 4:**
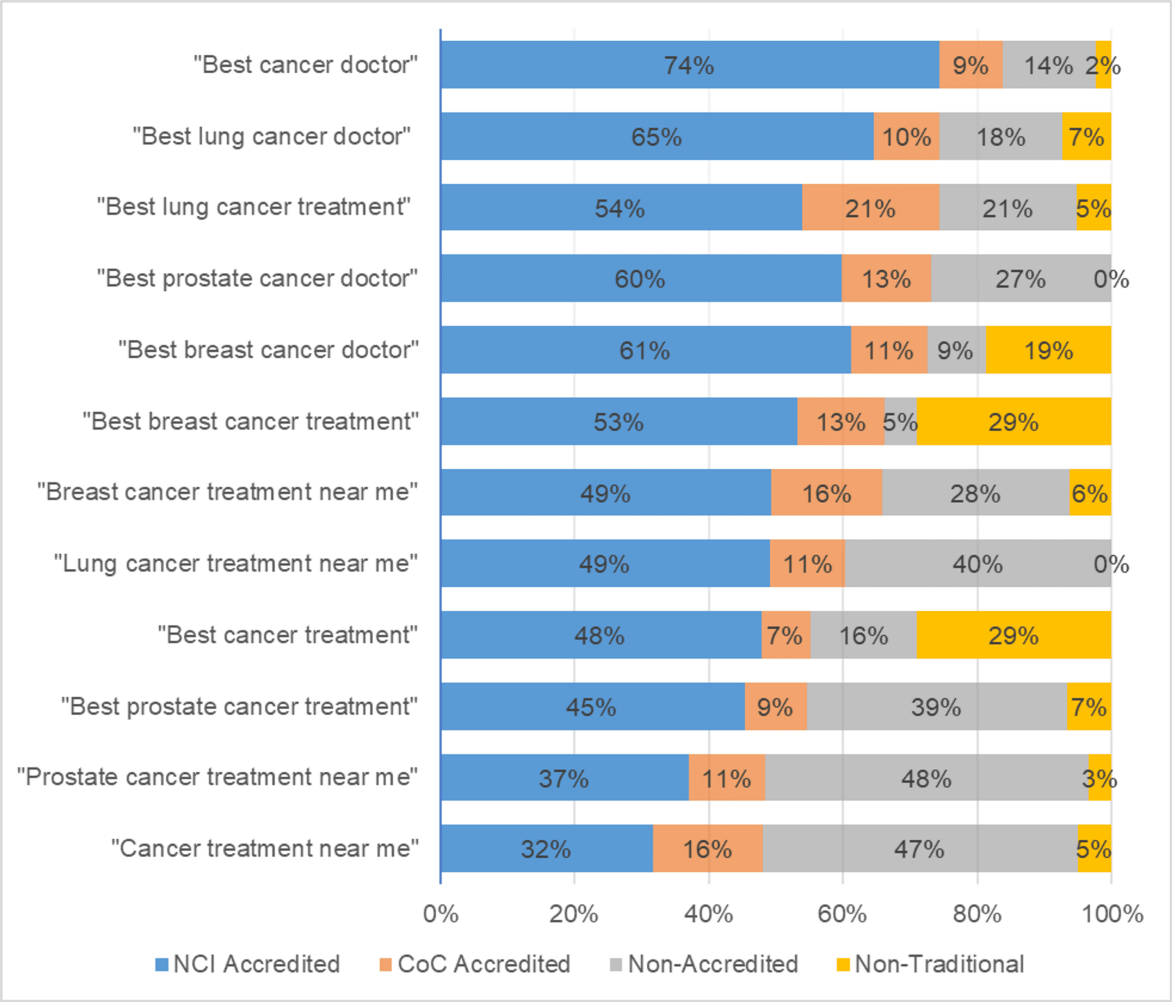
Percentage of search results that were National Cancer Institute (NCI)-accredited centers, Commission on Cancer (CoC)-accredited centers, non-accredited centers, and non-traditional centers based on search term used and sorted by the highest percentage of accredited centers

The search phrase that yielded the highest ratio of NCI centers was “best cancer doctor” at 74.4% and the phrase that yielded the lowest was “cancer treatment near me” at 31.7%. The proportions of search result categories based on search phrases are shown in Figure 4.

Of the known 71 NCI centers in the country, 42 (59.2%) did not have any advertisements. Of the 35 NCI centers located in one of the target cities, 12 (34.3%) did not have any advertisements. Notably, two specific NCI centers accounted for 49.5% of the NCI advertisements. The advertisements representing these two NCI centers included 80.2% of advertisement results for cities in which they do not have a physical presence. Similarly, one specific non-traditional treatment center accounted for 38 of the 79 non-traditional center advertisements (48.1%), and 35 of 38 of those advertisements (92.1%) were in cities in which this center had no physical presence. 

**Figure 5:**
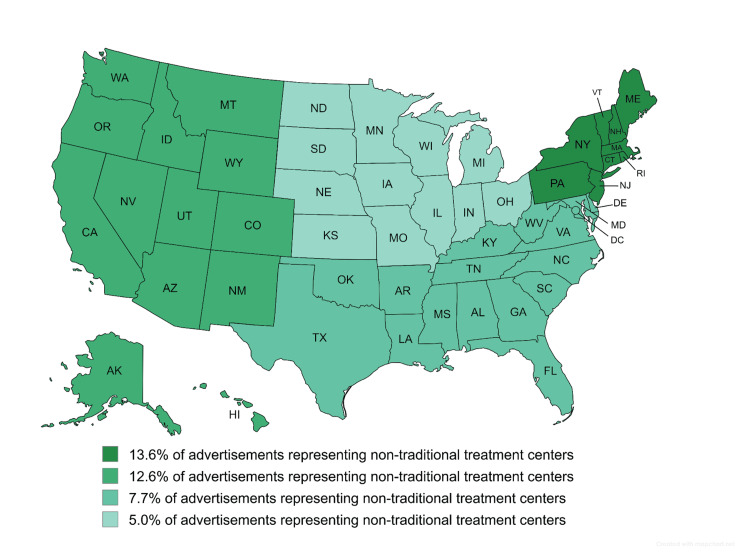
Ratio of non-traditional treatment center advertisements by region

Regarding non-traditional treatment centers, there was geographic variation in the results. Boston, San Francisco, Las Vegas, Oklahoma City, and San Jose had 38.1%, 23%, 23%, 24%, and 18% of searches yielding non-traditional treatment center advertisements, respectively. By contrast, six cities, i.e., Indianapolis, New York City, Jacksonville, Charlotte, Austin, and Columbus, showed no such advertisements. The ratio of non-traditional treatment center advertisements by region was as follows: Northeast with 13.6%, West with 12.6%, South with 7.7%, and Midwest with 5.0%, as shown in Figure 5. However, when excluding the center that accounted for 48.1% of non-traditional advertisements, the adjusted regional percentages were as follows: Northeast with 4.43%, West with 9.26%, South with 3.12%, and Midwest with 2.52%.

## Discussion

This study examined the variation of advertisements that appeared at the top of simulated Google searches pertaining to cancer treatment centers. Simulated searches were conducted from the most populous cities and the accreditation status of the treatment centers appearing in those results were analyzed. To our knowledge, this is the first study to do so. We uncovered a predominant presence of advertising for NCI-designated treatment centers in major cities such as Philadelphia, Seattle, and New York. Conversely, non-traditional treatment centers were the least common result type but had a higher percentage in certain regions (i.e. Northeast/West). Future research could investigate whether regional variations in advertisement types reflect differences in patient preferences or underlying local healthcare cultures. Studies have shown that acceptance of non-traditional medicine varies based on medical specialty [12], but there is less evidence exploring regional preferences in the U.S.

Moreover, we discovered that a small subset of treatment centers disproportionately dominate the advertisement spaces across multiple geographic regions. For example, advertisements from just two of the 71 NCI treatment centers accounted for nearly half of all NCI-related advertisements in the study, while 60% of NCI centers did not have any advertising. It has been shown that 59% of patients use the Internet for health and medical information [13] and may not understand the accreditation status or quality of centers that appear in rank lists in Google searches. This raises concerns that patients may inadvertently seek care at non-traditional centers that, despite their high visibility, obscure the fact that they do not always offer evidence-based, multidisciplinary treatments such as surgery, radiation therapy, or systemic therapy - omissions that have been associated with worse survival outcomes [14,15]. 

In general, healthcare advertising has significant differences from non-healthcare marketing. The U.S. Food and Drug Association (FDA) requires that any pharmaceutical company include the generic name, uses, and potential side effects of any drugs [16]. Unlike the pharmaceutical industry, DTC marketing by treatment centers is not regulated. Currently, the United States and New Zealand are the only countries, which allow DTC marketing for healthcare [17]. 

Furthermore, there are significant ethical implications of DTC marketing particularly when directed toward the vulnerable oncology patient population [18]. While these advertisements can empower patients by providing information, they also introduce complexities relating to transparency and the advertiser’s financial motives [5]. It is important to recognize that advertising’s primary aim is to attract patients frequently by leveraging prestige-driven or emotional tactics [5-7]. 

There are several limitations that need to be acknowledged. Internet searches can be confounded due to the personalization of search results, which are influenced by factors, such as IP address, search history, and browser type. To assess this potential bias, 40 additional searches were conducted using a different browser and including a privacy VPN. The results were compared with those obtained using the study’s standard search methods. The methods showed moderate agreement. This corresponds to a kappa value between 0.41 and 0.60, indicating that while there was some consistency between search results, notable variability remained, potentially influenced by factors such as search personalization, algorithmic differences, or temporal fluctuations in advertisements. Another limitation inherent to digital marketing studies is the dynamic nature of paid advertisement campaigns, which are constantly changing, and thus this study only represents the results of the sample from December 2022.

## Conclusions

This study contributes to the evolving discussion on digital DTC marketing in healthcare by providing insights into its presence in cancer treatment center marketing. By examining the accreditation status of advertised treatment centers, we illuminate patterns that may influence patient perceptions and potentially healthcare decisions. Future research is needed to further understand patient perspectives and decision-making processes influenced by these advertisements, as well as explore the associations between accreditation status and patient outcomes.
